# Association between Periodontitis and Hypertension among Adult Population in Rwanda

**DOI:** 10.3390/jcm13164722

**Published:** 2024-08-12

**Authors:** Agnes Gatarayiha, Zoe Brookes, Stephen Rulisa, Amanuel Kidane Andegiorgish, Léon Mutesa

**Affiliations:** 1 School of Dentistry, College of Medicine and Health Sciences, University of Rwanda, Kigali P.O. Box 3286, Rwanda; 2 Peninsula Dental School, Plymouth University, Plymouth PL4 8AA, UK; 3 School of Medicine and Pharmacy, College of Medicine and Health Sciences, University of Rwanda, Kigali P.O. Box 3286, Rwanda; 4 School of Public Health, College of Medicine and Health Sciences, University of Rwanda, Kigali P.O. Box 3286, Rwanda; 5 Centre for Human Genetics, School of Medicine and Pharmacy, College of Medicine and Health Sciences, University of Rwanda, Kigali P.O. Box 3286, Rwanda

**Keywords:** periodontitis, periodontal diseases, hypertension, adults, Rwanda

## Abstract

**Background/Objectives**: Mortality due to various non-communicable diseases, including hypertension, is increasing globally. Studies have reported that periodontitis, a chronic inflammatory disorder caused by oral pathogens, is a potential risk factor for hypertension. These pathogens can invade arterial walls, leading to vascular inflammation and endothelial dysfunction, which then increases the likelihood of developing hypertension. However, evidence of the association between periodontitis and hypertension remains limited. Therefore, the aim of this study is to determine whether periodontitis is associated with hypertension among adults in Rwanda. **Methods**: A cross-sectional study was carried out among 420 participants (hypertensive and non-hypertensive) at the University Teaching Hospital of Kigali (CHUK) and Ruhengeri Hospital in Rwanda. Periodontitis was assessed using clinical parameters: clinical attachment loss (CAL), bleeding on probing (BoP), and periodontal pocket depth (PDD). Hypertension was defined as a patient with a systolic or diastolic blood pressure (SBP/DBP) of ≥140/90 mmHg. Descriptive statistics, the Chi-square test, and logistical regression were performed using SPSS version 29 for statistical data analysis. **Results**: The prevalence of periodontitis was found to be 69.5% among hypertensive patients and 52.4% among non-hypertensive patients. Clinical attachment loss was 6.24 times (AOR = 6.24, 95% CI: 1.99–19.56) higher among hypertensive patients and the difference was significant (*p* = 0.001). Other periodontal parameters such as periodontal pocket depth and bleeding on probing showed a more significant association among hypertensive than non-hypertensive patients. **Conclusions**: Our study found a significant association between periodontitis and hypertension in Rwandan adults. However, further intervention studies are needed to explore causality and potential interventions.

## 1. Introduction

Hypertension, characterized by an increased systolic or diastolic blood pressure (SBP/DBP) threshold of ≥140/90 mmHg [1], is a public health concern; it is a risk factor for cardiovascular diseases (CVDs) and a significant cause of premature mortality worldwide [2,3,4]. Globally, statistical data indicate that the prevalence of hypertension was estimated to be between 30 and 45% among the adult population [5]. Although the information is scarce, the prevalence of hypertension is high in low- and middle-income countries (LMICs: 31.5%, 1.04 billion people) compared to higher-income countries (HICs; 28.5%, 349 million people) [3,6]. In Africa, the variance in the prevalence of hypertension ranges from 37% to 75% [7,8], with 21.2% of adults in Rwanda having an elevated blood pressure [9]. These figures align with those from other sub-Saharan African countries—20% in Ethiopia, 29.6% in Ghana, 28.6%% in Kenya, and 14.6% in Uganda—along with figures of 51.7% in Nigeria and 75.3% in South Africa [7,10,11,12,13,14]; this underscores a persistent public health challenge despite extensive prevention and treatment efforts.

Periodontitis is a widespread chronic inflammatory condition resulting from bacterial infections, which leads to the destruction of tooth-supporting structures and, ultimately, tooth loss if left untreated [15]. This is predominantly caused by inadequate oral hygiene, which can cause an oral dysbiosis of host microbiota, including Porphyromonas gingivalis, Treponema denticola, and Tannerela forsythia [16]. Periodontitis affects over 50% of the global population, and it is the sixth most prevalent disease, with 11.2% of people experiencing severe periodontitis [17,18]. In the United States, a National Health and Nutrition Examination Survey in 2009–2012 revealed that 46% of adults had periodontitis, with 8.9% experiencing severe periodontitis [19]. In Africa, the prevalence varies, with 42.2% in Ethiopia [20], 43.3% in Ivory Coast [21], 50% in South Africa [22], and significant rates in Rwanda where a national oral health survey revealed that 60% of participants had dental calculus and 34.2% had dental debris [23].

Emerging evidence points to vascular inflammation as a key driver of endothelial dysfunction, thereby contributing to the onset and progression of hypertension [24,25]. Additionally, periodontitis has been identified as a potential risk factor for hypertension with a subsequent complication for the landscape of cardiovascular diseases [26,27,28]. The linkage between periodontitis and hypertension has garnered attention through studies in the USA and Korea, which demonstrated a higher likelihood of hypertension among individuals with periodontitis [29]. Further research in the USA established a positive linear relationship between the severity of periodontitis and the odds of hypertension, highlighting the significance of this association [28,29]. A study conducted in Moyu, China, reinforced this connection, showing a significant correlation between periodontitis and hypertension, especially among those with severe periodontal conditions [30]. 

The potential linkage between periodontal disease and cardiovascular diseases has increasingly become a focal point of research due to periodontitis’ chronic inflammatory nature, systemic inflammation markers, and its contribution to endothelial dysfunction leading to hypertension [31,32]. Knowingly, more than half of the world’s adult population is affected by periodontal disease, which is implicated in roughly 50% of CVD hypertensive groups across both developed and developing countries [27,28,33]. A study in Cameroon highlighted an association between CVDs and periodontitis [34], emphasizing the need for further investigation into this relationship within different populations in Africa, including Rwanda. While this association has been extensively investigated in different regions, the scarcity of reports from Rwanda lends distinctive significance to this research, filling a notable gap in the existing literature.

Given the prevalence of hypertension and its role in exacerbating cardiovascular disease risks, which accounts for 31% of global deaths and 11.3% of deaths in sub-Saharan Africa [16,35,36], there is a need to ascertain whether periodontitis may lead to the onset of hypertension in low-income settings. Therefore, this study was undertaken to investigate the association between periodontitis and hypertension among adult people in two referral hospitals in Rwanda. Identifying a link could underscore the importance of managing periodontal diseases as a possible preventive measure for reducing hypertension, aligning with the Rwandan government’s strategies for reducing non-communicable diseases through medical interventions and lifestyle modifications [37].

## 2. Materials and Methods

### 2.1. Study Design and Population

Our study utilized an observational cross-sectional design, employing a quantitative approach to assess the association between periodontitis and hypertension among adult people aged at least 18 years and above. A total of 420 participants were included in the study, with 210 individuals diagnosed with hypertension and 210 individuals without hypertension. 

The study was carried out in two major referral hospitals in Rwanda. The first, the University Teaching Hospital of Kigali (CHUK), situated in the Nyarugenge District of Kigali, serves as a primary referral and teaching hospital, handling patients with hypertension referred from various parts of Rwanda. The second, Ruhengeri Hospital, located in the Musanze District of the Northern Province of Rwanda, caters to patients from the surrounding districts, including Burera and Gakenke, an area with a high prevalence of hypertension [38]. These hospitals were selected purposefully, leveraging the high prevalence of hypertension in Rwanda [39] to facilitate a comprehensive examination of the potential link between periodontitis and hypertension.

Eligible participants were recruited from the internal medicine department at the University Teaching Hospital of Kigali (CHUK) and the Non-Communicable Diseases Clinic at Ruhengeri Hospital from October 2023 to March 2024. 

One group included hypertensive patients aged 18 years and above, who consented to participate and had at least twenty teeth, regardless of undergoing anti-hypertensive treatment. Conversely, the other group comprised non-hypertensive patients attending outpatient services, excluding those from dental services who also provided their consent to participate in the study. However, the study excluded patients who were using dentures or orthodontic braces, pregnant women, those who had used antibiotics or mouthwash within the four weeks leading up to the study, or those unable to answer questions or who were non-cooperative. 

The sample size was determined using G* Power 3.1.9.7 Software, aiming to detect a specified effect size (0.25) with a standard error of 0.05 and a 95% power level, resulting in a total of 420 participants equally divided between the hypertensive group and the non-hypertensive group [40]. 

### 2.2. Data Collection Procedures

Data collection for this study was conducted by trained dental therapists using structured interviews to obtain information on the participants’ socio-demographic characteristics and dental and medical history, alongside clinical oral examinations to assess the periodontal status of the participants. 

Hypertension was defined based on established parameters: a systolic blood pressure (SBP) of 140 mmHg or higher and a diastolic blood pressure (DBP) of 90 mmHg or higher, as well as a consideration of patients currently taking anti-hypertensive medications [41,42]. Blood pressure measurements were conducted by nurses trained in using an upper arm automatic sphygmomanometer machine (OMRON M3). Patients were required to sit conformably for at least 5 min before the BP measurement and systolic and diastolic blood pressure (SBP and DBP) values were recorded and provided to the patients.

In the context of this study, periodontitis was identified through specific clinical criteria, including the presence of two or more interproximal sites with a clinical attachment loss (CAL) of 3 mm or greater (≥3 mm) or the presence of one or more interproximal sites with a pocket depth (PD) of 4 mm or greater (≥4 mm) on at least two or more different teeth [41,43,44]. A full-mouth clinical examination for determining periodontitis was carried out, considering clinical parameters such as bleeding on probing (BoP), periodontal pocket depth (PDD), and clinical attachment loss (CAL), which were measured using a University of North Carolina (UNC)-15 for both the hypertensive group and the non-hypertensive group.

Periodontal pocket depth was measured in millimeters (mm) as the distance from the gingival margin to the base of the pocket, and clinical attachment loss was measured in millimeters from the cement–enamel junction (CEJ) to the bottom of the pocket. These were both examined by calibrated examiners and assessed at six sites at the buccal, mesio-buccal, distal-buccal, lingual, mesio-lingual, and disto-lingual of all teeth excluding third molars. All the procedures were conducted with the support of dental therapists and nurses who had undergone rigorous week-long training and calibration to ensure consistent and reliable data collection. Moreover, the piloting process was conducted for the purpose of familiarization with and the adaptation of the questionnaires before the real data collection exercise. 

### 2.3. Statistical Analysis

The analysis of data was performed using SPSS (Statistical Package for the Social Sciences) Version 29 software. A descriptive analysis of the studied variables, including age, gender, hospital location, smoking status, alcohol consumption, and medical history, was performed and presented in terms of frequency and percentage.

The statistical significance of the differences was analyzed using the Chi-square test for dependent variables (hypertension) and other independent variables (periodontitis, socio-demographic characteristics, and medical and dental conditions). A univariate logistic regression analysis was performed to assess the strength of the association between hypertension (outcome) and each study variable and the results were reported as a crude odds ratio (COR) along with a 95% confidence interval. Building on the initial findings, a multiple logistic regression was performed to confirm the association between hypertension and periodontitis after adjusting the potential confounders, and the results were reported as an adjusted odds ratio (AOR) with a 95% confidence interval. Age, gender, smoking status, alcohol consumption, and the presence of chronic diseases were included as covariates in our statistical analyses to adjust for their potential confounding effect. Statistical considerations were meticulously addressed throughout the study, with all the tests being two-tailed, and *p*-values of <0.05 were considered statistically significant with the precision of the estimated odds ratios evaluated through the application of 95% confidence intervals (CIs).

Ethical clearance was obtained from the Institution Review Board of the University of Rwanda, College of Medicine, and Health Sciences (Reference No 486/CMHS IRB/2023), complemented by specific authorizations from both the University Teaching Hospital of Kigali (CHUK) and Ruhengeri Hospital. In alignment with these, all potential participants were provided with detailed information regarding the study and the participants voluntarily signed a consent form before the data collection. Moreover, this approach prioritized the confidentiality of participant information, safeguarding personal and sensitive data throughout the research process.

## 3. Results

In this study, we compared the socio-demographic characteristics of a non-hypertensive group (*n* = 210) and a hypertensive group (*n* = 210), focusing on sex, age, and geographical location. The hypertensive group had a higher proportion of females (55.7%) compared to the non-hypertensive group (51.4%), with no significant difference (*p* = 0.775). The average age was 48.58 years (SD = 14.77), with a significant difference between the hypertensive group and the non-hypertensive group (*p* < 0.001). Notably, the oldest age group (at least 55 years old) dominated the hypertensive group (54.8% (*n* = 115)) compared to the non-hypertensive group (19.1%). Younger participants (22–32 years old) were less prevalent in the hypertensive group (8.6%) compared to the non-hypertensive group (24.8%). According to the study location, most participants in the hypertensive group were from Ruhengeri Hospital (71.4%), whereas the majority of the participants in the non-hypertensive group were from the University Teaching Hospital of Kigali (CHUK) (63.3%), showing a significant difference (*p* < 0.001). These findings highlight the importance of age and location in the prevalence of hypertension. As shown in Table 1.

### Periodontal Diseases and Other Risk Factors Associated with Hypertension

Our analysis also indicated that the prevalence of periodontitis was significantly higher among hypertensive (69.5%) compared to non-hypertensive individuals (52.4%). 

As shown in Table 2, this study revealed a multifaceted landscape of risk factors associated with hypertension. Among the demographic factors, age significantly influenced hypertension risk. The odds of developing hypertension increased with age, with the oldest age group (≥55 years) exhibiting a crude odds ratio (COR) of 8.73 for hypertension (*p* < 0.001) and an adjusted odds ratio (AOR) of 4.29 (*p* = 0.004), indicating a strong association between advanced age and the likelihood of hypertension. Additionally, individuals from Ruhengeri Hospital had above a 4.3-fold increase in hypertension risk (COR = 4.32, AOR = 4.99, *p* < 0.001) compared to those from the University Teaching Hospital of Kigali (CHUK), highlighting geographical disparities in hypertension prevalence.

Notably, teeth cleaning was significantly linked to the likelihood of hypertension. The lack of teeth cleaning was associated with a more than threefold increase in risk (COR = 3.03, *p* < 0.001; AOR = 11, *p* < 0.001), underscoring the potential role of oral hygiene in modulating hypertension risk.

Moreover, individuals who currently smoked experienced a 4.10-fold increase in hypertension risk (COR = 4.10, *p* < 0.001), although this association did not reach statistical significance in the adjusted model (AOR = 2.8, *p* = 0.208). For alcohol consumption, individuals who consumed alcohol had lower odds of hypertension (COR = 0.44, *p* = 0.001; AOR = 0.415, *p* = 0.015), while those who stopped over 6 months ago also showed a reduced risk (COR = 0.70, *p* = 0.12; AOR = 0.47, *p* = 0.027).

Chronic diseases such as diabetes, cardiovascular diseases, and HIV/AIDS markedly influenced hypertension risk. Individuals with diabetes exhibited a significantly elevated risk, with both unadjusted and adjusted odds ratios indicating a substantial increase (COR = 1.46, *p* < 0.001; AOR = 7.22, *p* < 0.001). Cardiovascular diseases were associated with a more than threefold increase in hypertension risk (COR = 3.38, *p* = 0.02), although adjusted odds suggested a lesser association (AOR = 1.21, *p* = 0.77). Furthermore, individuals with HIV/AIDS had an elevated risk (COR = 2.70, *p* = 0.003; AOR = 1.55, *p* = 0.298).

Specific parameters within the periodontitis parameters, such as bleeding on probing, periodontal pocket depth, clinical attachment loss, and gingival recession were identified as closely associated with hypertension. Bleeding on probing showed a nearly fourfold increase in hypertension risk (COR = 3.88, *p* < 0.001; AOR = 2.76, *p* = 0.028) compared to individuals with no bleeding on probing. Pocket depth was also significant; individuals with a pocket depth of 5 mm or greater had a threefold increase in hypertension risk (COR = 3.076, *p* < 0.001; AOR = 1.95, *p* = 0.098), although the adjusted model did not reach statistical significance. For clinical attachment loss, those with clinical attachment loss equal to or less than 3 mm had an increased risk of hypertension (COR = 2.074, *p* < 0.001; AOR = 6.24, *p* < 0.011), and those with a CAL of 6 mm or more exhibited a higher risk (COR = 2.597, *p* < 0.001; AOR = 2.40, *p* = 0.036). Gingival recession also showed a significant influence, where individuals with gingival recession had a more than threefold increase in hypertension risk (COR = 3.51, *p* < 0.001; AOR = 4.71, *p* = 0.010). 

## 4. Discussion

The findings from this study provide compelling evidence linking periodontitis to hypertension among the adult population in Rwanda. This association underscores the multifactorial nature of hypertension, involving demographic factors, lifestyle choices, health conditions, and, particularly, oral health. The study included two groups, a hypertensive patient group with 210 participants and another normotensive group with 210 participants. The prevalence of periodontitis was significantly higher among hypertensive patients (69.5%) compared to non-hypertensive patients (52.4%). These findings align with other studies that have shown a higher prevalence of periodontal diseases among hypertensive individuals compared to their normotensive counterparts [45,46,47].

Findings from this study showed a strong association between periodontitis and hypertension, where periodontal parameters such as bleeding on probing, periodontal pocket depth, clinical attachment loss, and gum recession were found to have significantly higher odds among hypertensive patients. These results are consistent with previous research that has identified periodontal disease as a potential risk factor for hypertension [16,48].

The underlying mechanisms linking periodontal disease to hypertension may include systemic inflammation, endothelial dysfunction, and the dysregulation of the immune response. Periodontal pathogens can enter the bloodstream, thereby triggering systemic inflammatory responses that contribute to vascular changes and hypertension [49]. These inflammatory responses are mediated by cytokines such as interleukin-6 (IL-6) and tumor necrosis factor-alpha (TNF-α), which disrupt endothelial function, impair vasodilation, and increase vascular resistance, contributing to elevated blood pressure. Additionally, periodontal disease may lead to increased oxidative stress and reduced nitric oxide availability, further exacerbating hypertension [50].

On the other hand, age emerged as a significant predictor of hypertension, with the risk increasing substantially in older age groups. This finding aligns with previous studies that have consistently shown age as a critical risk factor for hypertension [51]. The progressive increase in blood pressure with age could be attributed to structural and functional changes in the vascular system, such as arterial stiffness and endothelial dysfunction [52]. The study location disparity observed, with patients from Ruhengeri Hospital exhibiting a higher hypertension risk compared to those from the University Teaching Hospital of Kigali (CHUK), highlights potential differences in healthcare access, socioeconomic status, and lifestyle factors between regions. Similar disparities have been documented in other studies, indicating that regional variations can significantly impact hypertension prevalence and management [53]. However, the location disparity in hypertension prevalence may also be influenced by demographic variables, such as age differences in the cohorts from each hospital. This should be considered when interpreting the findings.

This study’s findings on smoking and alcohol consumption provide nuanced insights into lifestyle factors influencing hypertension. Patients with a history of smoking had a 4.10 times higher risk of developing hypertension compared to non-smokers in the current study. This is consistent with a study conducted in Ethiopia by Bonsa et al. [54], which demonstrated that smoking was a significant factor associated with hypertension. While smoking was associated with increased hypertension risk, this association did not reach statistical significance in the adjusted model, suggesting the need for further investigation into smoking’s role in hypertension within this population. Conversely, alcohol consumption was inversely associated with hypertension, which contrasts with the established understanding that excessive alcohol intake increases blood pressure [55,56]. This paradoxical finding might be explained by the specific drinking patterns and types of alcohol and quantity consumed in the Rwandan context, warranting further research.

The significant association between chronic diseases such as diabetes and cardiovascular diseases and hypertension aligns with the well-established link between these conditions. Diabetes and cardiovascular diseases exacerbate hypertension through various mechanisms, including insulin resistance, inflammation, and vascular changes [57]. The observed elevated risk of hypertension in individuals with HIV/AIDS is also noteworthy, given that HIV infection and its treatment are known to contribute to cardiovascular risk [58].

Despite the robust findings, this study has limitations. The sample size of 420 individuals is a main hurdle, as it does not facilitate conducting more subgroup analyses without losing statistical power. This limitation may affect the generalizability of the findings and should be considered in future studies. Seasonal variations in blood pressure levels were not accounted for, which should be addressed in subsequent research. While this study is limited to Rwanda, the findings could potentially apply to other sub-Saharan countries with similar demographic and health profiles, highlighting the need for further research in these settings. Additionally, the study design limits causal inferences, and, therefore, longitudinal studies are needed to establish causal relationships between periodontal disease and hypertension.

Furthermore, this study did not specifically examine the interaction between anti-hypertensive medication and periodontal health, particularly drug-induced gingival proliferation. Future studies should address this interaction to provide a more comprehensive understanding of the relationship between anti-hypertensive medications and periodontal conditions in the context of hypertension. Moreover, future research could explore preliminary genetic associations between periodontitis and hypertension using publicly available Genome-Wide Association Studies (GWAS) databases and Mendelian randomization analysis, providing a direction for future mechanistic research. Interventional studies could provide valuable insights into whether improving periodontal health can directly reduce hypertension risk. Additionally, factors like smoking and geographical location that are linked to both periodontitis and hypertension may obscure the relationship that has been established. Future investigations should also consider interventional studies examining the effects of periodontal therapies on hypertension.

## 5. Conclusions

This study underscores the significant association between periodontal disease and hypertension among the adult population in Rwanda. Individuals with periodontal disease, including indicators such as bleeding on probing, periodontal pocket depths, clinical attachment loss, and gingival recession, have significantly higher odds of developing hypertension. These findings align with previous research and highlight the multifactorial nature of hypertension, involving systemic inflammation, endothelial dysfunction, and immune response dysregulation triggered by periodontal pathogens. This understanding integrates oral health as a significant contributing factor to hypertension pathophysiology.

Practically, these findings suggest that integrating dental care into routine health assessments and public health strategies could be crucial in addressing the burden of hypertension and improving cardiovascular health. Public health initiatives should improve access to dental care and promote oral hygiene, particularly in low- and middle-income countries. Educational campaigns highlighting the link between oral health and systemic diseases like hypertension can raise awareness and encourage proactive health behaviors.

## Figures and Tables

**Table 1:** Socio-demographic characteristics.

		Non-Hypertensive Group	Hypertensive Group	*p*-Value
		Count (%)	Count (%)	
Gender	Male	102 (48.6%)	93 (44.3%)	0.775
	Female	108 (51.4%)	117 (55.7%)	
Age	22–32 years	52 (24.8%)	18 (8.6%)	<0.001
	33–43 years	73 (34.8%)	37 (17.6%)	
	44–54 years	47 (22.8%)	40 (19.0%)	
	≥55 years	38 (19.1%)	115 (54.8%)	
Location of participant	University Teaching Hospital of Kigali (CHUK)	133 (63.3%)	60 (28.6%)	<0.001
	Ruhengeri Hospital	77 (36.7%)	150 (71.4%)	
Smoking status	Yes	4 (1.9%)	14 (6.7%)	<0.001
	Never smoked	192 (91.4%)	164 (78.1%)	
	Stopped over 6 months ago	14 (6.7%)	32 (15.2%)	
Alcohol consumption	Yes	73 (34.8%)	46 (21.9%)	0.004
	Never	62 (29.5%)	89 (42.4%)	
	Stopped over 6 months ago	75 (35.7%)	75 (35.7%)	
Presence of diabetes	Yes	25 (11.9%)	123 (58.6%)	<0.001
	No	185 (88.1%)	87 (41.4%)	
Cardiovascular diseases	Yes	5 (2.4%)	16 (7.6%)	0.014
	No	205 (97.6%)	194 (92.4%)	
Having HIV/AIDS	Yes	14 (6.7%)	34 (16.2%)	0.002
	No	196 (93.3%)	176 (83.8%)	

Note: CHUK: University Teaching Hospital of Kigali.

**Table 2:** Periodontal diseases and risk factors associated with hypertension.

Predictor Variables	COR	95% C.I.	*p*-Value	AOR	95% C.I.	*p*-Value
**Age of participant**						
22–32 years	Ref [1.00]			Ref [1.00]		
33–43 years	1.464	0.75–2.85	0.26	1.058	0.41–2.74	0.88
44–54 years	2.459	1.24–4.86	<0.01	1.362	0.52–3.57	0.52
≥55 years	8.743	4.56–16.74	<0.001	4.290	1.59–11.54	0.004
**Location**						
Ruhengeri Hospital	4.32	2.86–6.51	<0.001	4.99	2.73–9.27	<0.001
University Teaching Hospital of Kigali (CHUK)	Ref [1.00]			Ref [1.00]		
**Smoking status of participants**						
Yes	4.10	1.32–12.69	<0.001	2.88	0.55–15	0.208
Stopped over 6 months ago	2.68	1.38–5.20	0.010	0.52	0.209–1.274	0.151
Never smoked	Ref [1.00]			Ref [1.00]		
**Alcohol consumption**						
Yes	0.44	0.27–0.72	0.001	0.415	0.20–0.84	0.015
Stopped over 6 months ago	0.70	0.44–1.10	0.12	0.47	0.24–0.92	0.027
Never	Ref [1.00]			Ref [1.00]		
**Having Diabetes**						
Yes	1.46	6.35–17.25	<0.001	7.23	3.73–14	<0.001
No	Ref [1.00]			Ref [1.00]		
**Cardiovascular diseases**						
Yes	3.38	1.21–9.41	0.02	1.51	0.40–5.62	0.545
No	Ref [1.00]			Ref [1.00]		
**Having HIV/AIDS**						
Yes	2.70	1.40–5.21	0.003	1.32	0.55–3.19	0.538
No	Ref [1.00]			Ref [1.00]		
**Cleaning teeth**						
No	3.03	1.77–5.20	<0.001	11.063	4.78–25.58	<0.001
Yes	Ref [1.00]			Ref [1.00]		
**Periodontitis parameters**						
**Bleeding on probing (BoP)**						
Yes	3.88	2.55–5.93	<0.001	2.76	1.12–6.61	0.028
No	Ref [1.00]			Ref [1.00]		
**Pocket Depth (PD)**						
PD ≥ 5 mm	3.076	1.72–5.50	<0.001	1.95	0.89–4.91	0.098
PD ≤ 4 mm	Ref [1.00]			Ref [1.00]		
**Clinical attachment loss (CAL)**						
Less than or equal to 3 mm	0.601	0.39–0.92	0.01	6.24	1.99–19.56	<0.011
6 mm and above	2.597	1.39–4.84		2.40	1.06–5.44	0.036
4–5 mm	Ref [1.00]			Ref [1.00]		
**Gingival recession**						
Yes	3.51	2.30–5.34	<0.001	4.71	1.14–15.28	0.010
No	Ref [1.00]			Ref [1.00]		

Note: COR: Crude Odds Ratio, AOR: Adjusted Odds Ratio.

## Data Availability

The data presented in this study are available upon request from the corresponding author due to privacy or ethical restrictions.
